# Supplementary material for: Reconfigurations within resonating communities of brain regions following TMS reveal different scales of processing
Source: Netw Neurosci. 2020 Jul 1;4(3):611–36. doi: 10.1162/netn_a_00139 (PMC7462427; doi:10.1162/netn_a_00139)
Supplement: Supplementary file 1 [file netn-04-611-s001.pdf]

Garcia, J. O., Ashourvan, A., Thurman, S. M., Srinivasan, R., Bassett, D. S., & Vettel, J. M. (2020).
Supporting information for "Reconfigurations within resonating communities of brain regions following TMS reveal different scales of processing." *Network Neuroscience*, *4*(3), 611–636.
https://doi.org/10.1162/netn_a_00139

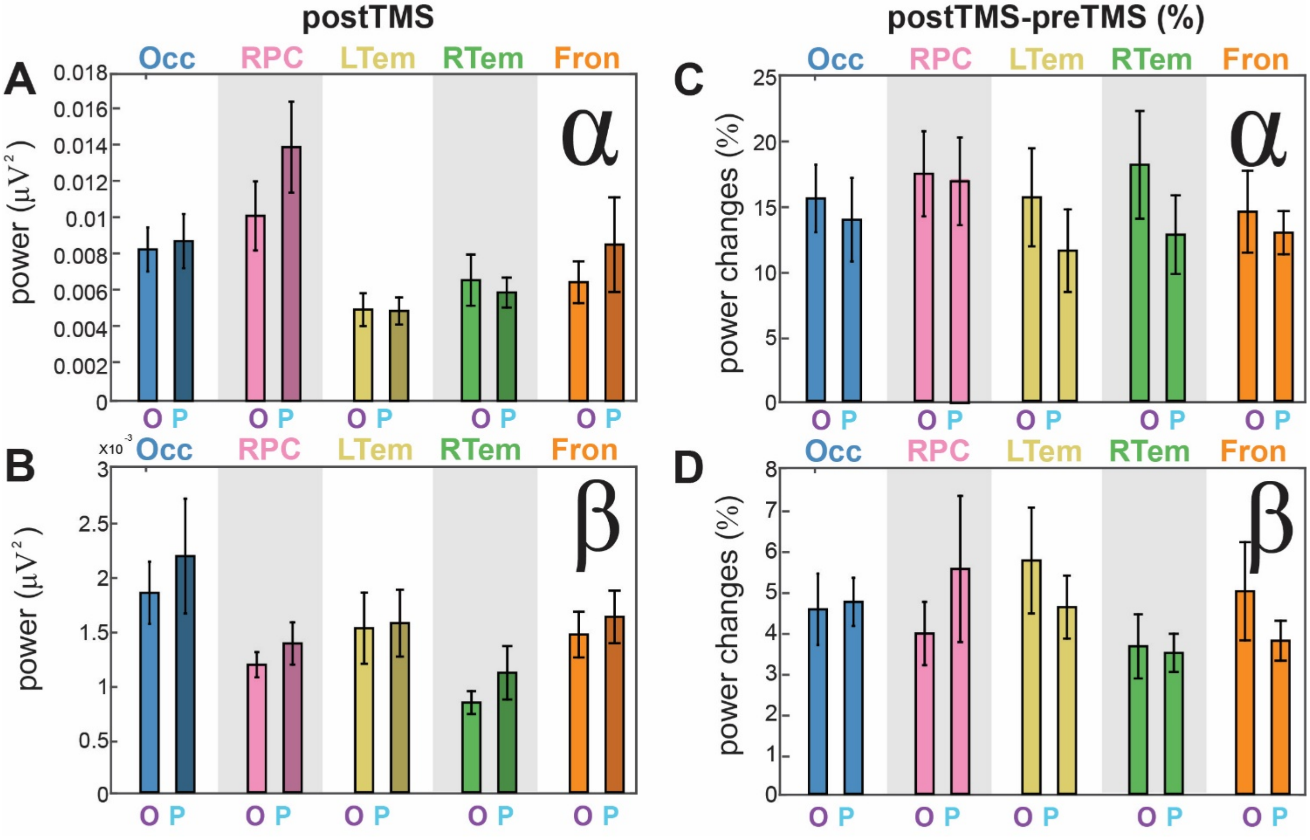

Supplemental Figure 1: Average power and power changes in communities for occipital (O) and parietal (P) stimulation for the alpha band (A,C) and beta band (B,D). (A,B) Power estimated from the second following stimulation and averaged across nodes within a community and then across participants Error bars indicate the SEM across participants. (C,D) Power changes plotted as percent change from the preTMS level of power. Error bars indicate the SEM across participants

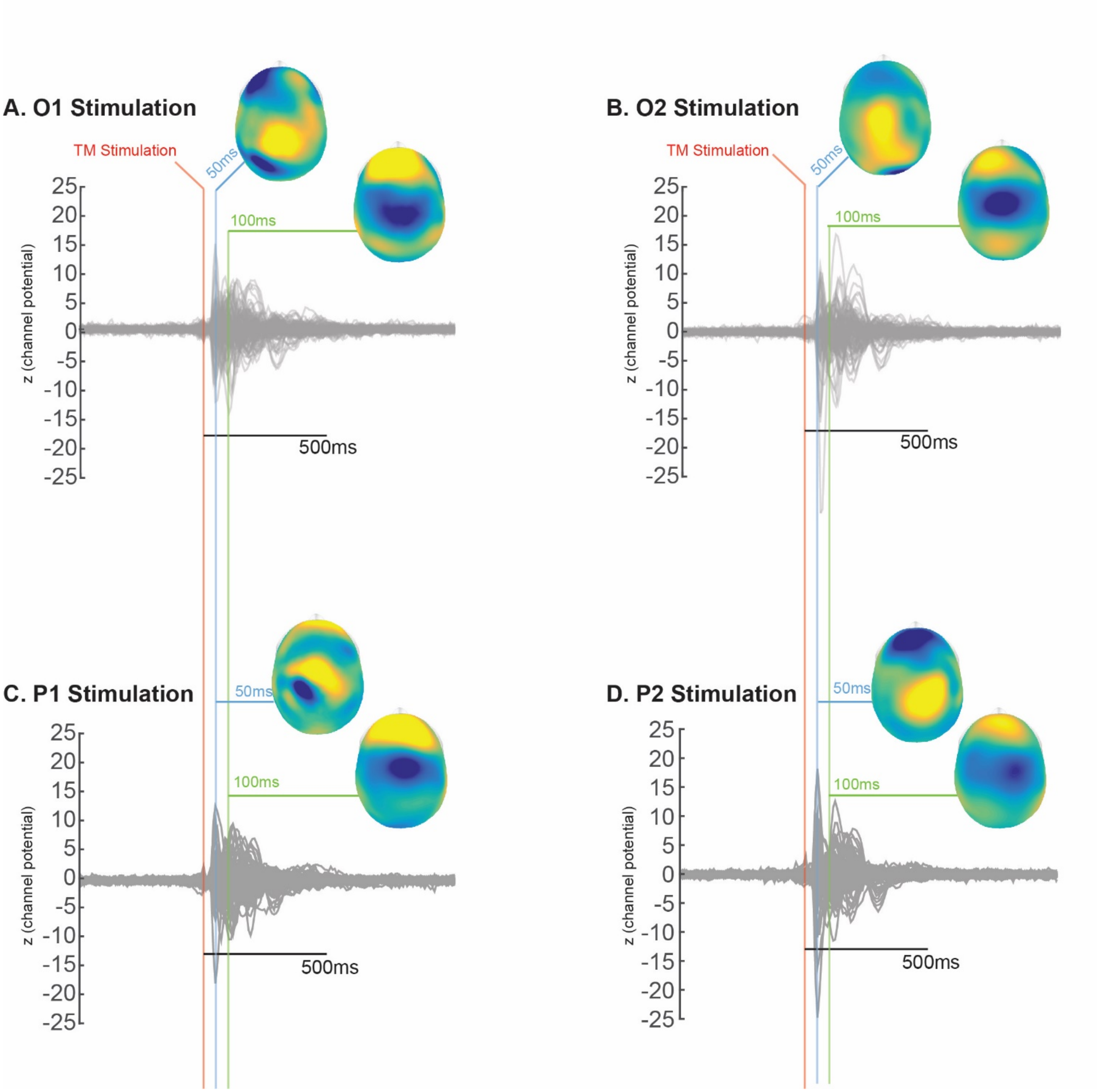

Supplemental Figure 2: Mean channel traces (normalized by the mean and standard deviation of 500ms of samples before TM stimulation) across participants with topographic plot insets visualizing the amplitude across all 128 channels showing increases (yellow) and decreases (blue) across the scalp. (A,B) Mean evoked response to left (A) and right (B) occipital stimulation show clear and obvious changes near the stimulation site 50ms after stimulation (blue line) and a subsequent general negative deflection 100 ms after stimulation, typical of single pulse TEPs. (C,D) Mean evoked response to left (C) and right (D) parietal stimulation display a similar pattern to occipital stimulation, with a spatial shift anterior to the peaks shown with occipital stimulation.

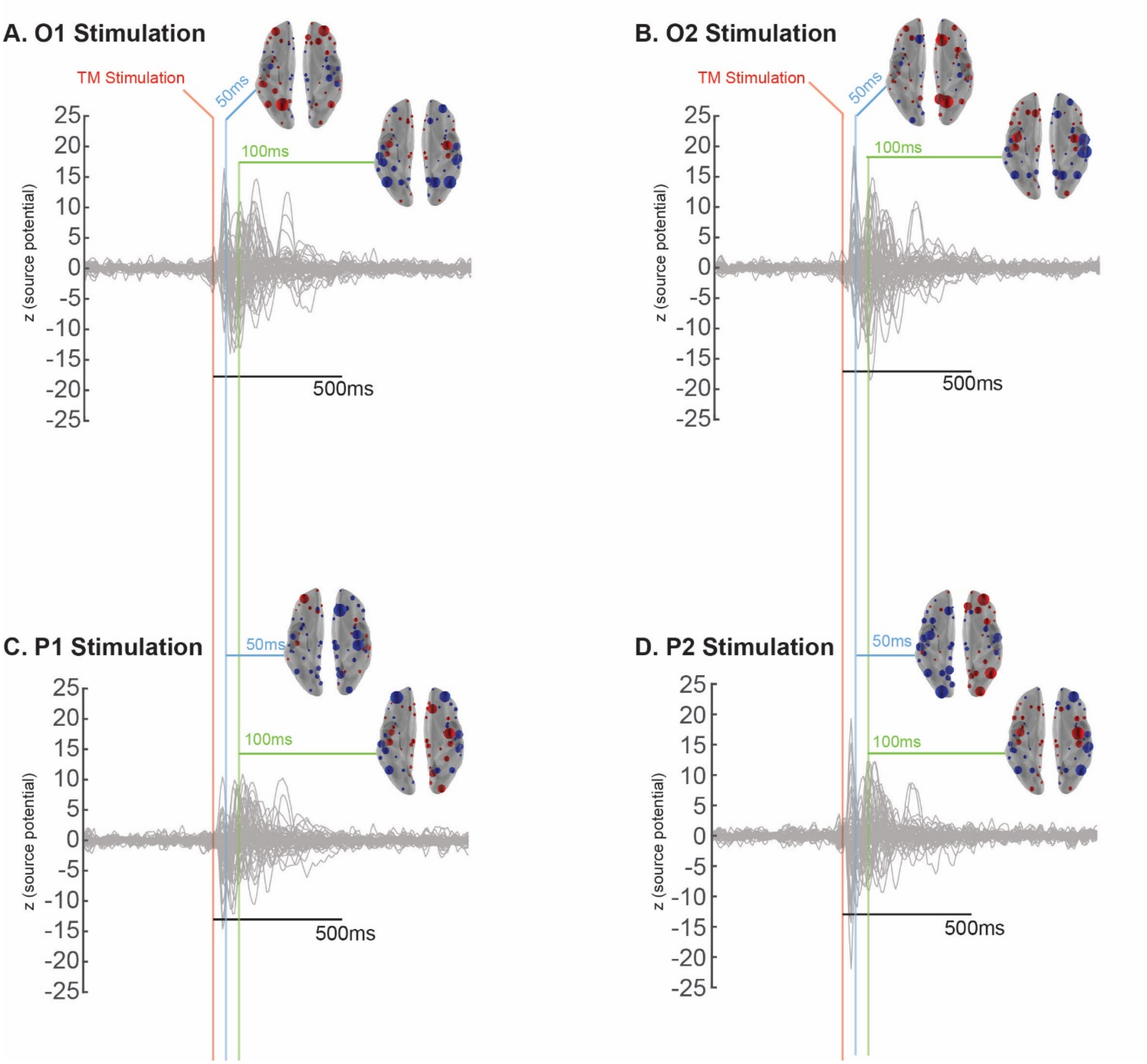

Supplemental Figure 3: Mean source potentials (normalized by the mean and standard deviation of 500ms of samples before TM stimulation across participants with brain insets visualizing the source potential across all 68 regions of the brain (scaled within each time slice). (A,B) Mean evoked source potential to left (A) and right (B) occipital stimulation show a clear enhancement near the stimulation site 50ms after stimulation (blue line) and a subsequent reversal 100 ms after stimulation, typical of single pulse TEPs. (C,D) Mean evoked source potential to left (C) and right (D) parietal stimulation display slightly more variability for left stimulation, but the standard enhancement followed by reversal for right stimulation.

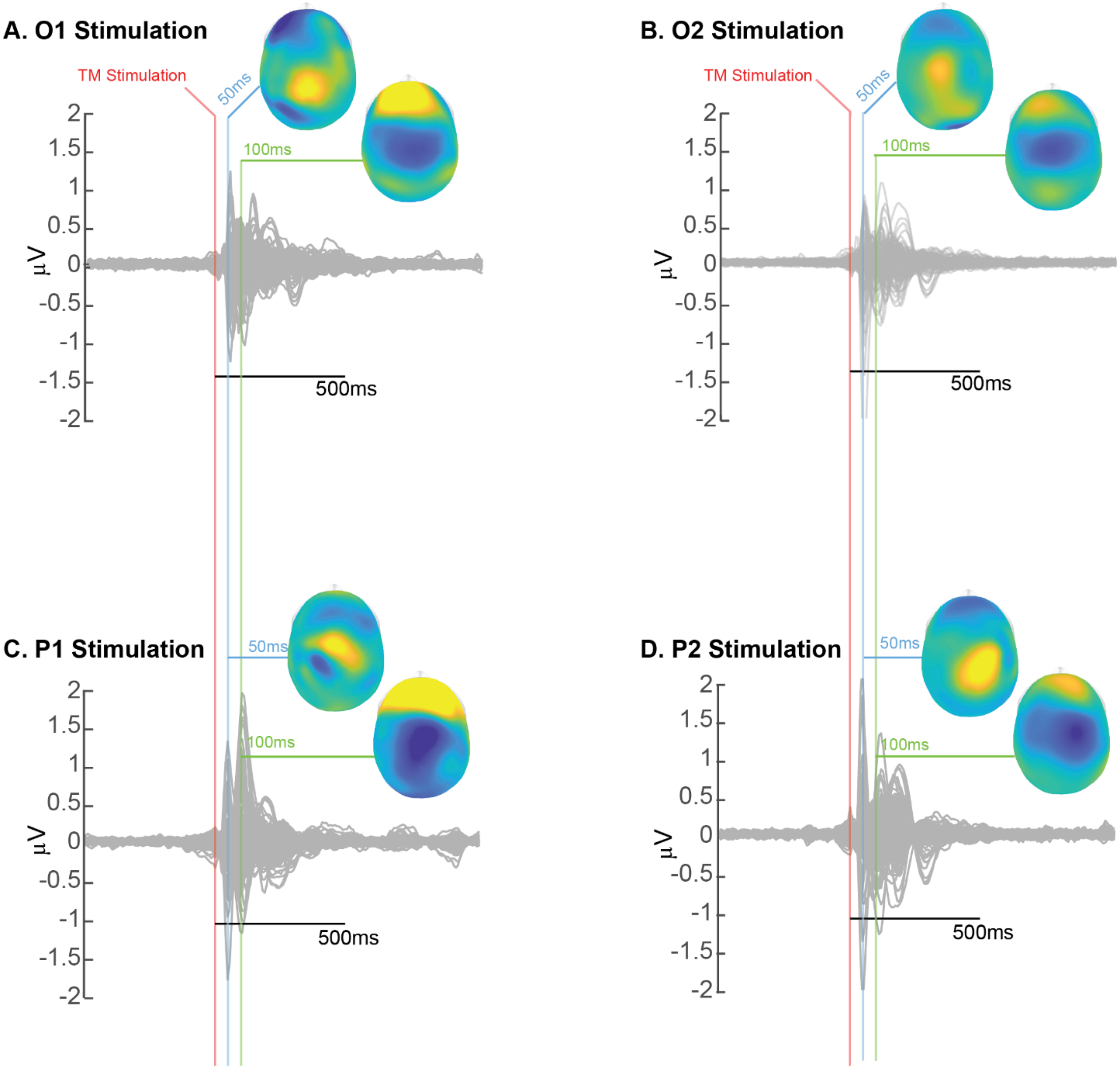

Supplemental Figure 4: Mean channel traces (with baseline subtraction 500ms of samples before TM stimulation) across participants with topographic plot insets visualizing the amplitude across all 128 channels showing increases (yellow) and decreases (blue) across the scalp. (A,B) Mean evoked response to left (A) and right (B) occipital stimulation show clear and obvious changes near the stimulation site 50ms after stimulation (blue line) and a subsequent general negative deflection 100 ms after stimulation, typical of single pulse TEPs. (C,D) Mean evoked response to left (C) and right (D) parietal stimulation display a similar pattern to occipital stimulation, with a spatial shift anterior to the peaks shown with occipital stimulation.

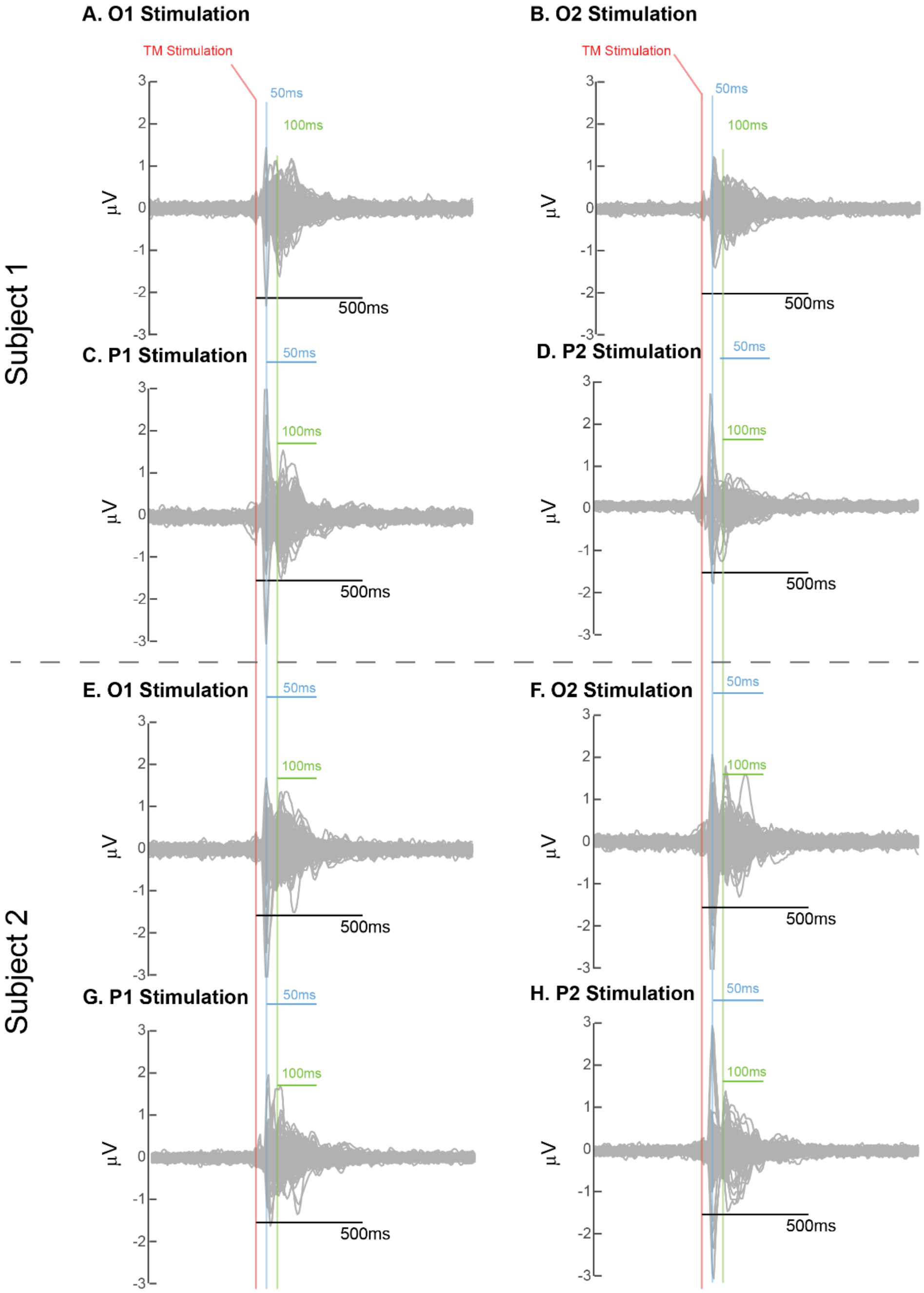

Supplemental Figure 5: Mean channel traces (with baseline subtraction 500ms of samples before TM stimulation) within two sample subjects, highlighting the consistency in timing of the evoked responses, but also the small changes in amplitude across participants. (A,B,E,F) Mean evoked response to left (A) and right (B) occipital stimulation show clear and obvious changes near the stimulation site 50ms after stimulation (blue line) and a subsequent general negative deflection 100 ms after stimulation, typical of single pulse TEPs. (C,D,G,H) Mean evoked response to left (C) and right (D) parietal stimulation display a similar pattern to occipital stimulation.

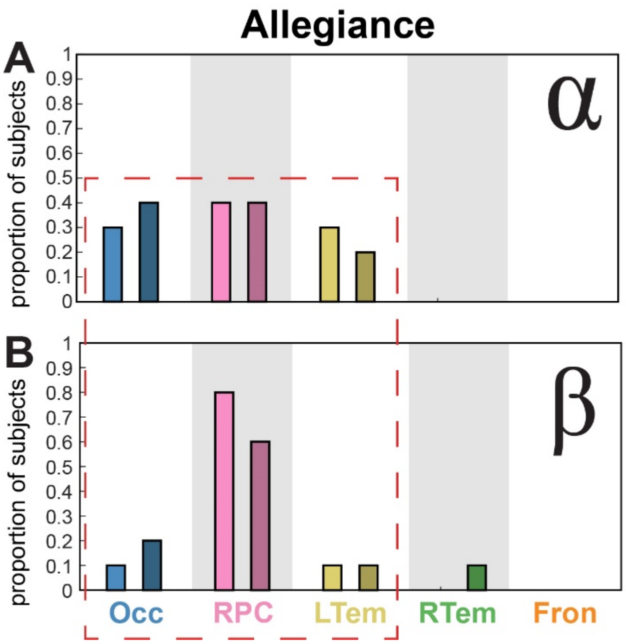

Supplemental Figure 6: Bar plots show the proportion of subjects who have the highest allegiance (A,B) and flexibility (C,D) in each frequency band, providing some evidence of robustness of the results. Highlighted in red, estimated allegiance in 6 and 8 subjects in the RPC community is the highest in the beta band for occipital and parietal stimulation, respectively. In contrast, estimated allegiance in the 3 communities closest to the stimulation site is similarly robust across these communities.

	**Allegiance**		**Flexibility**	
Rho(p)	*Alpha*	*Beta*	*Alpha*	*Beta*
Occipital	-.70(.23)	-.10(.95)	.30(.68)	.70(.23)
Parietal	**-.80(.13)**	**-.80(.13)**	.40(.52)	.30(.68)

Supplemental Figure 7: Spearman's Rho non-parametric correlation analysis correlating Allegiance and Flexibility with distance from the stimulation site. The analysis reveals opposing trends (negative *Rho* for allegiance and positive *Rho* for flexibility), but none of the results were significant (all $p > .05$). Parietal stimulation showed the strongest relationship, as indicated by the largest magnitude in Rho and the lowest p-value ($p = 0.13$). This analysis confirms much of what we see in the data; however, the non-significant effects do not change our interpretation.

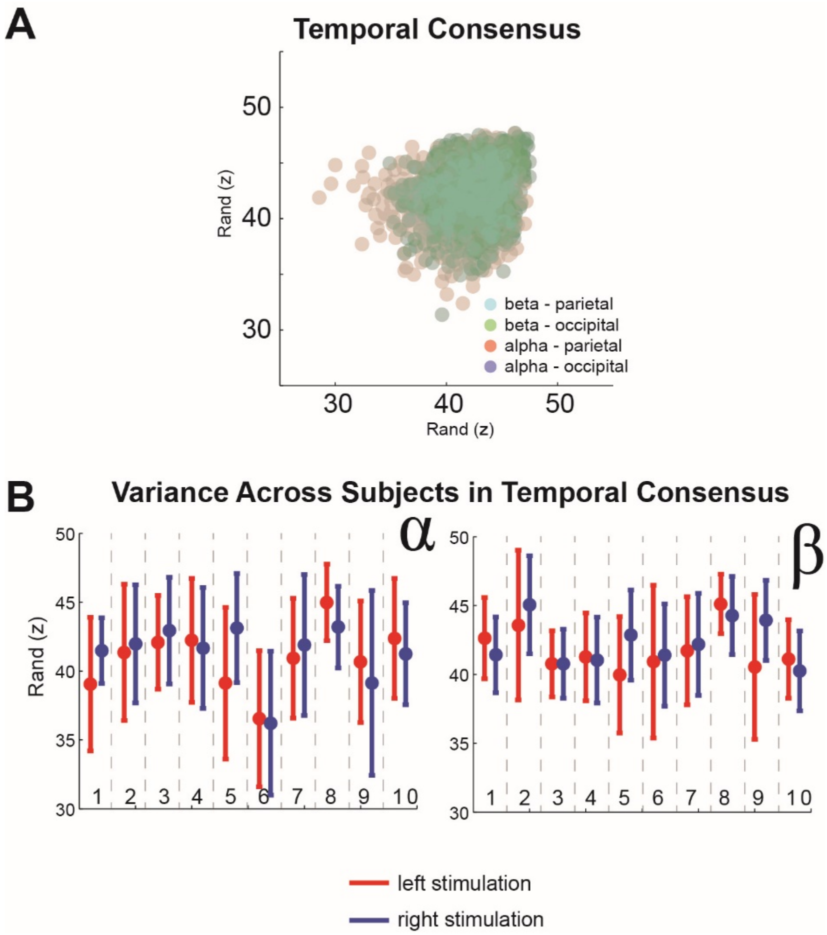

Supplemental Figure 8: Left and right stimulation similarity found with the Rand index, a metric of similarity between clusters (A) We plot the Rand z-score for each application of the community detection algorithm (100 iterations for each of 10 subjects), where each semi-transparent dot is colored to indicate stimulation condition (parietal or occipital) and frequency band (alpha or beta). Across all participants, only a very narrow range of Rand z-scores is observed, with high variability between all conditions. Due to this difference across conditions and iterations, we examined the variance within each subject across the 100 iterations within each condition. (B) Rand z-scores for alpha are in the left plot and beta in the right plot with scores for stimulation to right hemisphere in blue and left hemisphere in red. The error bars indicate the 95% confidence of the mean estimate. As illustrated in the plot, each subject's distributions demonstrate narrow overlapping ranges, where between-subject variability is much more substantial than the left-right stimulation difference.
